# The Role of Autophagy in Sepsis: Protection and Injury to Organs

**DOI:** 10.3389/fphys.2019.01071

**Published:** 2019-08-23

**Authors:** Xin Yin, Huang Xin, Shuai Mao, Guangping Wu, Liheng Guo

**Affiliations:** Department of Critical Care Medicine, The Second Clinical College of Guangzhou University of Chinese Medicine, Guangzhou, China

**Keywords:** autophagy, sepsis, immunity, oxidative stress, ncRNAs

## Abstract

Sepsis is a systemic inflammatory disease with infection, and autophagy has been shown to play an important role in sepsis. This review summarizes the main regulatory mechanisms of autophagy in sepsis and its latest research. Recent studies have shown that autophagy can regulate innate immune processes and acquired immune processes, and the regulation of autophagy in different immune cells is different. Mitophagy can select damaged mitochondria and remove it to deal with oxidative stress damage. The process of mitophagy is regulated by other factors. Non-coding RNA is also an important factor in the regulation of autophagy. In addition, more and more studies in recent years have shown that autophagy plays different roles in different organs. It tends to be protective in the lungs, heart, kidneys, and brain, and tends to be damaging in skeletal muscle. We also mentioned that some drugs can regulate autophagy. The process of modulating autophagy through drug intervention appears to be a new potential hope for the treatment of sepsis.

## Introduction

Sepsis is a “global health problem” that threatens human health and consumes a lot of health resources. It has the characteristics of high morbidity, high mortality, and high treatment costs. According to a recent epidemiological survey by the Intensive Care Over Nations (ICON), the incidence of sepsis varies from 13.6 to 39.3% in different regions, and intensive care unit (ICU) and hospital mortality in patients with sepsis are 25.8 and 35.3%. Sepsis is the main cause of morbidity in modern intensive care units, which imposes a considerable burden on modern ICUs. The most common source of infection for sepsis is the respiratory tract (67.4%) followed by the abdomen (21.8%), with positive strains having been retrieved in 69.6% of septic patients and Gram-negative microorganisms having been isolated from 67.1% of patients (Sakr et al., 2018). Although early diagnosis and treatment options have been developed for sepsis, there has been no significant reduction in overall mortality. Global epidemiological data can help raise awareness of sepsis and provide important information for future prevention and treatment.

Sepsis is a systemic inflammatory response syndrome caused by infection. It is clinically confirmed by the presence of bacteria or by highly suspicious infections. Further development may cause the dysfunction of multiple organs (Rhodes et al., 2017). The mechanism involves complex systemic inflammatory network effects (Balk, 2014), genetic polymorphisms (Arcaroli et al., 2005), immune dysfunction (Delano and Ward, 2016), coagulopathy (Simmons and Pittet, 2015), tissue damage, and abnormal responses of the host to different infectious pathogenic microorganisms and their toxins (Minasyan, 2019). Organ pathophysiological changes are closely related.

Recently, the mechanism of action of autophagy in sepsis has received increased attention. Autophagy is an evolutionarily conserved degradation system in cells and is involved in establishing an intracellular homeostasis. The process of autophagy includes autophagosomal formation, autophagosome-lysosome fusion, and the degradation products (Klionsky et al., 2016). In sepsis, autophagy has long been recognized as a cellular adaptive protective mechanism that limits cell damage and apoptosis (Karagiannidis et al., 2016). Autophagy not only eliminates damaged proteins and organelles, but also eliminates bacteria and pathogens present in the cytoplasm (Oami et al., 2018). Although some special bacteria such as *Staphylococcus aureus* can evade selective autophagy by activating host cell kinases, autophagy still plays an essential role in sepsis (Neumann et al., 2016). In the early stage of sepsis, the occurrence of autophagy and high levels of cell viability should represent the cytoprotective mechanism against microbial infection. However, this benefit is limited. When severe sepsis occurs, even a significant increase in autophagy does not reverse the overwhelming inflammatory response. In later stages of the disease, autophagy reflects the efforts of the cells to adapt to lethal conditions rather than the mechanism of cell death. Therefore, “autophagic cell death” is an inaccurate expression. Although inflammation and necrosis increase, cell death may be the result of other lethal signal cascade activations (Karagiannidis et al., 2016).

In this review, we describe the regulation of autophagy and its mechanism of action in sepsis. We also describe the manifestations of autophagy in different organ failures in sepsis. In addition, it is necessary to further identify potential therapeutic targets in the autophagy mechanism.

## Regulation of Autophagy in Sepsis and its Mechanism of Action

### Autophagy and Immune Regulation

The immune mechanisms of sepsis include innate immunity and adaptive immunity. The immune process involves the interaction of various immune cells and any defect will lead to the inhibition of immune function. Immunosuppression plays a crucial role in the pathogenesis of sepsis, either with the initial over-activation of the inflammatory response or with delayed immunosuppression after excessive inflammatory response (Molinaro et al., 2013; Lewis et al., 2016).

**Figure 1:**
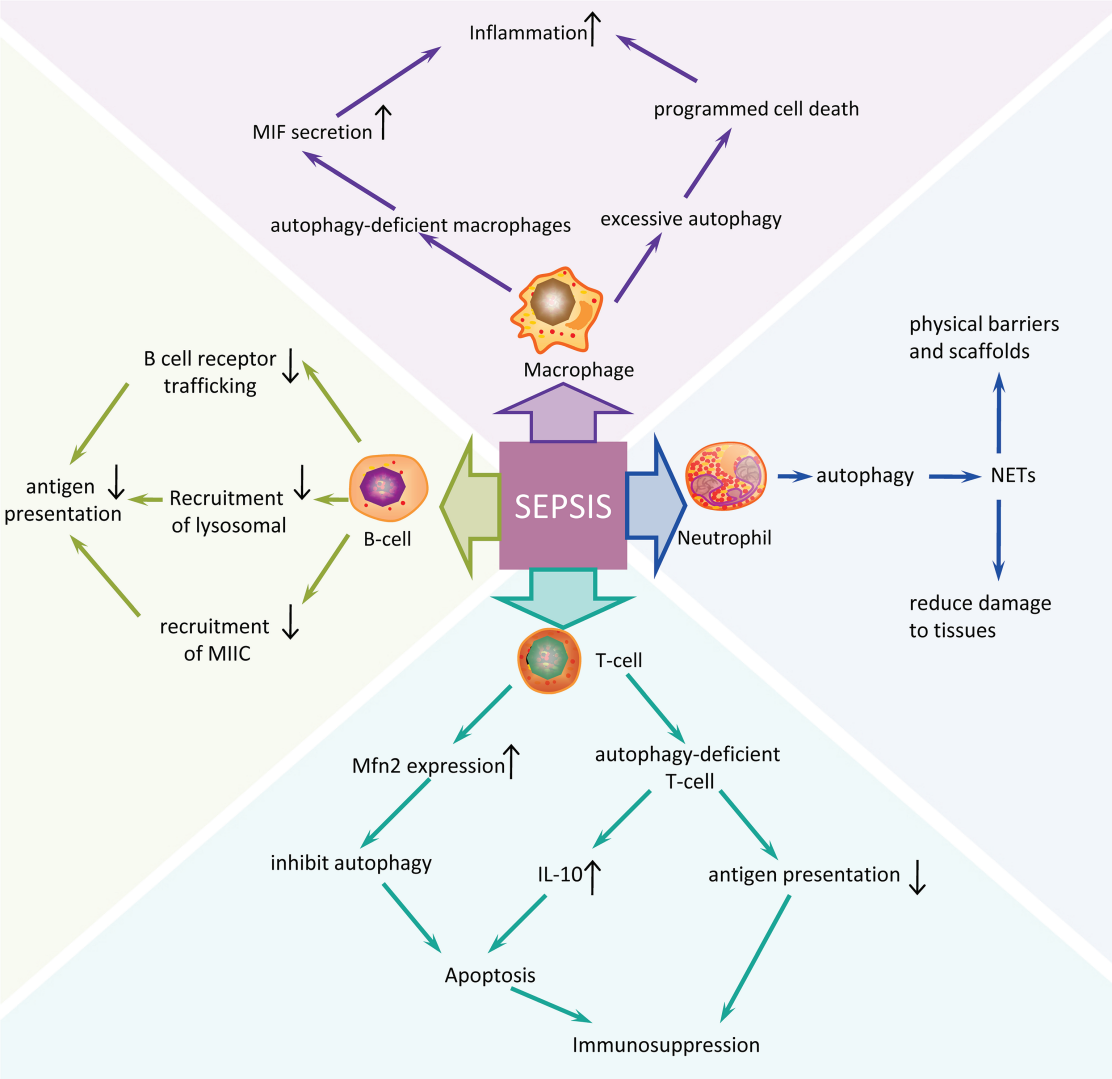
Autophagy regulation mechanism of different immune cells in sepsis. An overview of many possible mechanisms in innate immune and acquired immune cells may contribute to the pathogenesis of sepsis. Sepsis is a very serious systemic infectious disease by which severity is closely related to the host’s immune function. The postulated mechanisms by which different immune cells respond to sepsis might contribute to the development of sepsis are based on studies *in vitro* and animal models. The induction of autophagy in sepsis often promotes the immune response process of immune cells; while inhibition of autophagy or autophagy defects aggravates the inflammatory response and immunosuppression of sepsis. NETs, neutrophil extracellular traps.

The innate immune system is the first line of defense against infection in sepsis (Figure 1). This includes macrophages, neutrophils, dendritic cells, and natural killer (NK) cells. Autophagy has been shown to be closely related to the innate immune system, which can alleviate excessive inflammatory responses (Giegerich et al., 2014). One study found that autophagy-deficient macrophages using lipopolysaccharide (LPS) intervention could cause an increase in macrophage migration inhibitory factor (MIF) secretion and aggravate inflammation (Lee et al., 2016). However, excessive autophagy can also lead to the programmed cell death of macrophages, further exacerbating the inflammatory response (Qiu et al., 2019). In addition, neutrophils isolated from patients surviving sepsis showed an increase in autophagy induction, and induction of autophagy triggered neutrophil formation of neutrophil extracellular traps (NETs) and vice versa. NETs, through the extracellular chromosome skeleton, form a physical barrier and scaffold to contain microbes and enhance the synergy of antibacterial agents, reducing damage to host tissues and are one of the important defenses of the body’s natural immune system (Park et al., 2017). Acquired immunity is primarily a defense function against specific pathogens. It is mainly involved in the role of T cells and B cells. Apoptosis of CD4+ T cells is the main pathophysiological mechanism of immune function inhibition in the pathogenesis of sepsis. Mitofusin 2 (Mfn2) is a complete mitochondrial outer membrane protein and has been shown to be a negative regulator of autophagy. During sepsis, Mfn2 expression is increased which could inhibit autophagy and increase apoptosis of CD4+ T cells, thereby suppressing immune function (Ying et al., 2017). Another study also reached the same conclusion. Studies have found that CD4+ T cells in sepsis model mice had insufficient autophagy, though autophagosomes were increased. Autophagy was blocked by ATG5 knockout, and the production of interleukin-10 was significantly increased in CD4+ T cells along with an increase in apoptosis (Oami et al., 2016). Autophagy defects can reduce antigen presentation by T cells leading to immunosuppression. Studies have found that in the case of ATG5 deficiency, autophagy was reduced and antigen presentation by homologous T cells was also reduced, leading to the occurrence of immunosuppression (Neumann et al., 2016). Autophagy proteins are also an important factor in controlling B cell terminal activation steps. ATG5 are involved in B cell receptor trafficking and recruitment of lysosomal and MHC class II-enriched compartment (MIIC) in polarized B cells, which is essential for the acquisition and presentation of particular antigens. In conclusion, autophagy plays a role in immune regulation by regulating the immune response process of various immune cells in sepsis.

### Mitophagy and Oxidative Stress

**Figure 2:**
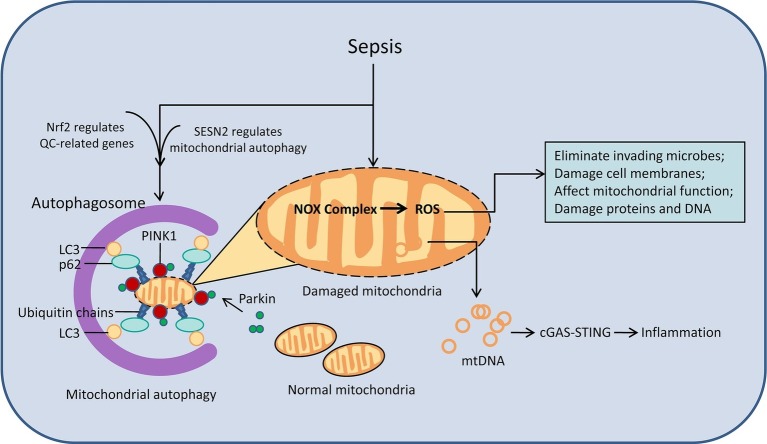
Mitophagy and oxidative stress in damaged mitochondria. After the onset of sepsis, the number of damaged mitochondria increases. Damaged mitochondria cause mitochondrial dysfunction through ROS signaling pathways and mtDNA, causing a series of damage. Autophagy can remove damaged mitochondria. The process of autophagy is regulated by cytokines such as Nrf2 and SESN2. cGAS, cyclic GMP-AMP synthase; mtDNA, mitochondrial DNA; QC, quality control system.

Mitochondrial dysfunction is currently considered to be an important factor in organ failure caused by sepsis and may impair cellular energy supply and increase oxidative stress (Figure 2; Thiessen et al., 2017). After microbial infection, the NADPH oxidase (NOX) complex in phagocytes and neutrophils produces reactive oxygen species (ROS) in a mitochondria-independent manner, which is essential for the body to eliminate invading microbes (Lambeth, 2004). In many and perhaps all cells, mitochondria are still the main source of ROS production (Kim et al., 2016b). Mitochondrial destruction is a potential source of cellular damage, and the release of mitochondrial damage-related patterns (such as mitochondrial DNA) drives innate immune responses and can lead to further oxidative stress damage. Excessive ROS can directly damage cell membranes, affect mitochondrial function, and damage proteins and DNA (Ghanta et al., 2017). Autophagy can select damaged mitochondria to be removed by ATG-dependent or independent pathways (Ding and Yin, 2012; Chang et al., 2015), and finally promote the renewal of damaged proteins and organelles through lysosomal-dependent degradation pathways to cope with oxidative stress damage caused by ROS excess (Baechler et al., 2019).

Mitophagy is part of the mitochondrial quality control (QC) system, which ingests dysfunctional mitochondria and then degrades it. Mitochondria are prone to damage in sepsis and the inner membrane potential of mitochondria decreases. The PINK1 protein accumulates on the outer mitochondrial membrane and then Parkin is recruited onto mitochondria and is activated by PINK1. The increased activated Parkin then builds ubiquitin chains on damaged mitochondria to tag them for degradation. The ubiquitin chain binds to the autophagic protein receptor, which binds to LC3 on the autophagosome, to induce mitophagy (Sun et al., 2018, 2019). Identification of damaged mitochondria and their assembly into autophagosomes requires increased expression of certain redox-sensitive proteins and direct targeting of mitochondria, and the SQSTM1/p62 protein is one of the key proteins in this process. The autophagic receptor protein SQSTM1/p62 acts to recognize ubiquitinated mitochondrial proteins and link them, through binding to LC3 to autophagosome (Preau et al., 2016). Damaged mitochondria are isolated by autophagosomes and eventually degraded by fusion with lysosomes, promoting the recovery of septic organ function (Bartz et al., 2015; Pan et al., 2018). Another key transcription factor for mitochondrial quality control is nuclear factor erythroid 2-related factor 2 (Nrf2) (Stępkowski and Kruszewski, 2011). Activation or inhibition of autophagy directly affects Nrf2 expression and nuclear translocation, and the affected genes include mitochondrial quality control-related genes (Zuo et al., 2019).

Impaired autophagy in mitochondria results in reduced mitochondrial clearance and an increase in inflammation. Studies have shown that sestrin 2 (SESN2), a stress-inducing protein, inhibits NLR family pyrin domain containing 3 protein (NLRP3) inflammasome activation by inducing mitophagy in macrophages to clear damaged mitochondria. SESN2 knockout mice showed impaired mitophagy and sepsis model mice showed excessive activation of the inflammatory body and increased mortality (Kim et al., 2016a). Interferon gene stimulator (STING) is a transmembrane protein located in the endoplasmic reticulum membrane or mitochondrial outer membrane. Exogenous DNA present in the cytoplasm can cause abnormal activation of STING, leading to increased inflammation and cell death. In sepsis, the fusion of autophagosomes and lysosomes is inhibited, and autophagy damage leads to decreased degradation of damaged mitochondria. It has been demonstrated that mtDNA liberation following mitochondrial damage, activates the cGAS–STING pathway and induces a cascade of inflammatory responses (Kemp, 2017; Prabakaran et al., 2018; Hu et al., 2019). However, it is important to note that mitophagy defects in certain organs, such as the liver, are not the cause of organ or cell damage during sepsis. Studies have found that the degree of macrophage and neutrophil infiltration and the level of pro-inflammatory cytokines are not affected by the reduction of autophagy in the liver of autophagy-deficient sepsis model mice. Mitochondrial number, mitochondrial adenosine triphosphate (ATP), and oxidative stress levels were not affected by the loss of autophagy in hepatocytes (Lalazar et al., 2016).

In conclusion, mitophagy is of great significance in the fight against oxidative stress during the development of sepsis.

### Non-coding RNA Regulates Autophagy in Sepsis

Non-coding RNA refers to RNA that does not encode a protein. As research has progressed, the role of non-coding RNA has become well-known to researchers. Among them, miRNA, lncRNA, and circRNA can participate in a variety of biological processes and play important regulatory functions. When sepsis occurs, many non-coding RNAs play a role in regulating autophagy. The autophagy process regulated by ncRNA can directly act on autophagy-related proteins and indirectly regulate the signaling pathway of autophagy (Table 1). Beclin-1 is a key autophagy-promoting gene with products involved in the early stages of autophagosome formation. The study found that MiR-142a-5p targeted the 3′-UTR of Beclin-1 mRNA to inhibit its protein translation in damages of pulmonary endothelial cells (PECs). In septic lung injury, transplantation of mesenchymal stem cells (MSCs) can attenuate sepsis acute lung injury (ALI) by down-regulating miR-142a-5p and enhancing autophagy (Zhou and You, 2016). ATG12 directly promotes phagocytic elongation and autophagosome maturation, and can be targeted by miR-23a. In sepsis, down-regulation of miR-23a mitigates the inhibition of autophagy, leading to the suppression of inflammatory mediator (Si et al., 2018). The study found that over-expression of miR-335-5p induced autophagy by activating the AMPK/ULK1 (AMP-activated protein kinase/unc-51 like autophagy activating kinase 1) signaling pathway and attenuating the inflammatory response in a mouse model of sepsis (Gao et al., 2018). In LPS-treated human fibroblasts, nuclear factor kappa B 3 (NFκB3) was shown to be a functional target of miR-100, and overexpression of miR-100 inhibited LPS-induced damage to human fibroblasts (Liu et al., 2018a). Both AMPK and peroxisome proliferator-activated receptor α (PPARα) are autophagy-related signals and positively regulate autophagy. The expression of AMPK/PPARα and neighbor of BRCA1 lncRNA 2(NBR2) are regulated by miR-19a. miR-19a negatively regulates hepatocyte autophagy by LPS intervention through the modulation of AMPK/PPARα and NBR2 (Liu et al., 2018b). In a mouse model of sepsis, overexpression of miR-300 augmented autophagy by targeting nicotinamide phosphoribosyltransferase (NAMPT), activating the AMPK signaling pathway, and inhibiting cell apoptosis (Li et al., 2018). miR-155 attenuated inflammation of septic lung injury in mice and cell models by inhibiting autophagy induced by transforming growth factor-β-activated binding protein 2 (TAB2) (Liu et al., 2017b). Antagonization of miR-21-3p was able to organize myocardial mitochondrial ultrastructure damage and autophagy in LPS-treated mice, and miR-21-3p also controls sepsis-associated cardiac dysfunction by regulating SH3 domain-containing protein 2 (SORBS2) (Wang et al., 2016b). lncRNA is a non-coding RNA greater than 200 nts in length. It binds to transcription factors and interferes with its binding to the gene promoter region, thereby regulating transcription. It can also be used as a molecular sponge to adsorb miRNA and inhibit its binding to mRNA, so that mRNA is not degraded. By modulating miR-100, HAGLR opposite strand lncRNA (HAGLROS) inhibits apoptosis and autophagy in human fibroblasts induced by LPS (Liu et al., 2018a). In hepatocytes treated with LPS, NBR2 knockdown reverses the anti-autophagy effect of miR-19a inhibitors and leads to a decrease in LC3-II/LC3-I ratio and Beclin-1 expression, inhibiting autophagy to protect liver cells (Liu et al., 2018b).

**Table 1:** Summary of recent studies on non-coding RNAs that directly or indirectly regulate autophagy.

NcRNA	Organs or cell line	Response to autophagy	Target	References
**Direct**				
miR-142a-5p	Lung	Suppressed	Beclin-1 mRNA	Zhou and You, 2016
miR-23a	Macrophage cell	Suppressed	ATG12	Si et al., 2018
**Indirect**				
miR-335-5p	Lung	Induced	FASN	Gao et al., 2018
miR-100	Lung	Induced	NFκB3	Liu et al., 2018a
miR-19a	Liver	Suppressed	AMPK; PPARα	Liu et al., 2018b
miR-300	Liver	Induced	NAMPT	Li et al., 2018
miR-155	Lung	Induced	TAB2	Liu et al., 2017a
miR-21-3p	Heart	Induced	SORBS2	Wang et al., 2016a
lncRNA HAGLROS	Lung	Suppressed	miR-100	Liu et al., 2018a
lncRNA NBR2	Liver	Induced	miR-19a	Liu et al., 2018b

## Mechanism of Action of Autophagy in Different Organs

**Figure 3:**
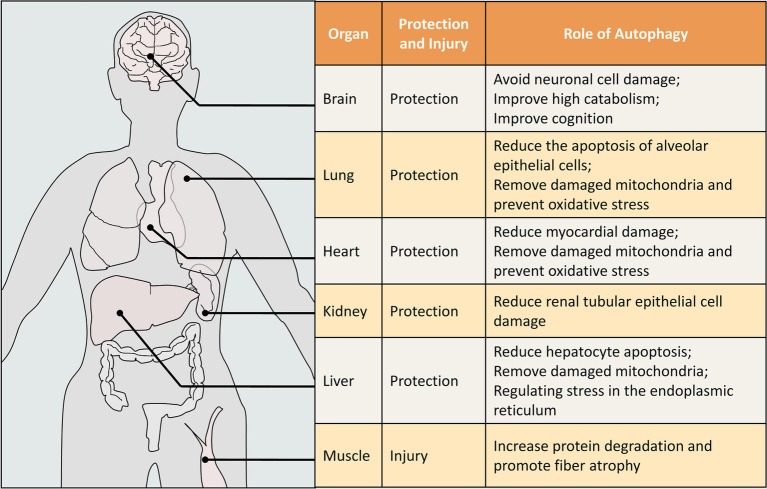
The role of autophagy in different organs of sepsis. This figure is illustrated for humans, but the data is obtained from humans and other animals (mouse, rat, etc.). The protective or damaging effect of autophagy on organs in sepsis is our comprehensive judgment after literature analysis.

In addition, more and more studies in recent years have shown that autophagy plays different roles in different organs. It tends to be protective in the lungs, heart, kidneys, and brain, and tends to be damaging in skeletal muscle (Figure 3).

### Autophagy and Sepsis-Induced Acute Lung Injury

Sepsis lung injury is one of the most common clinical complications of sepsis, manifested by the release of a large number of inflammatory factors, along with an increase in apoptosis and pulmonary edema. Current research indicates that excessive apoptosis of pulmonary epithelial cells plays an essential role in the pathogenesis of ALIs. It was reported that the induction of autophagy *via* rapamycin could protect alveolar epithelial cells from LPS-induced apoptosis, and vice versa. Therefore, we believe that LPS-induced apoptosis can be counteracted by elevated cellular autophagy (Ji et al., 2018). *In vivo* experiments also found that PICK1 deficiency caused a disruption of autophagy progress and amplified the damage in sepsis-induced ALIs. Lysosomal damage caused by PICK1 deficiency may be responsible for the impediment of autophagy flux (Mo et al., 2018). Another experiment found that the AMPK activator might improve lung recovery through the activation of mitochondrial biogenesis *via* the increased expression of PGC-1α. Treatment with the AMPK activator also further increased the expression of autophagy-associated proteins, suggesting that the increase of autophagy may be an additional protective mechanism in mitigating sepsis-induced lung injury (Kitzmiller et al., 2018). Increased autophagy in mitochondria also exerts lung protection. At 6 h after cecal ligation plus puncture (CLP) in mice, Nrf2 protein levels were significantly increased and alveolar mitophagy was activated. Elimination of damaged mitochondria by selective autophagy exerts cytoprotective effects in type 2 epithelium and alveolar macrophages (Chang et al., 2015). Therefore, we are more inclined to believe that autophagy plays a protective role in sepsis-induced lung injury.

### Autophagy and Sepsis-Induced Cardiac Dysfunction

Sepsis-induced cardiac dysfunction is an important component of multi-organ failure in sepsis. It manifests as a result of the inhibition of systolic and diastolic function, increased markers of myocardial damage, increased cardiac apoptosis, and destruction of myocardial mechanisms (Tsolaki et al., 2017). Most current studies suggest that induction of autophagy in sepsis can improve sepsis-induced myocardial damage. Rapamycin kinase (mTOR) is a key protein that inhibits the autophagy pathway. A recent study has shown that blocking the mTOR pathway had a protective effect on CLP-induced sepsis cardiac dysfunction, and this effect was mediated by the acceleration of autophagy (Han et al., 2018). Another study found that activation of AMPK can ameliorate myocardial contractile function in a mouse model of sepsis. Enhancing AMPK expression can increase autophagy *via* decreasing phosphorylation of mTOR and S6 (Zhang et al., 2017). In cardiomyocytes, transcription factors are an important factor in the regulation of autophagy. One study found that transgenic mice lacking IκBβ *via* S313 mutations were resistant to LPS-induced myocardial injury. Its mechanism of action is that hypophosphorylation of Iκ-Bβ can increase the translocation of NF-κB p65 into the nucleus in the cytoplasm. The promoter element of the autophagy-related gene Beclin-1 contains an NF-κB binding site, and NF-κB binds to the promoter of Beclin-1, resulting in their transcriptional activation and autophagy (Wang et al., 2016a; Zhou et al., 2017). The transcription factor EB (TFEB) is one of the regulators of autophagy, and it translocates to the nucleus and regulates the “coordinating lysosomal expression and regulation (CLEAR) network” genes, which are involved in autophagosome formation, vesicle formation, and extension, as well as substrate identification and degradation. Another study suggested that, compared to young mice, myocardial injury in aged septic mice was more severe because of the decrease in TFEB-mediated autophagy (Li et al., 2016a). Inhibition of the fusion process of autophagosomes and lysosomes also affects the integrity of the autophagic flux. Rubicon is thought to inhibit autophagosome maturation by negatively regulating vacuolar protein sorting 34 (Vps34) activity. Studies have shown that autophagy fluxes in Rubicon-deficient hearts were enhanced, and Rubicon knockout mice had improved diastolic filling and stroke volume increases (Zi et al., 2015).

As the heart is highly dependent on abundant ATP levels to maintain its function, impaired mitochondrial function is lethally detrimental to the septic heart. In order to maintain basic cellular function, repair or replacement damage in the face of such damaged mitochondria is vital. Mitophagy is one of the most important methods of self-repair. Mitophagy is part of an integrated mitochondrial quality-control system that takes dysfunctional mitochondria into the lysozyme and then degrades it. Prompt removal of damaged mitochondria can prevent oxidative damage. Otherwise, oxidative damage may cause apoptosis, leading to worsening of myocardial damage in sepsis (Pan et al., 2018). Beclin-1 promotes mitochondrial biogenesis *via* PINK1/Parkin as well as AMPK/ULK1, and also selectively facilitates PINK1-Parkin mitophagy for the clearance of damaged mitochondria. However, under septic challenge, other signals and the overwhelming inflammatory responses aggravate the deterioration of cardiac function when the signal of Beclin-1 is not sufficiently available (Sun et al., 2018, 2019). One interesting study found that fasudil improved LPS-induced mitochondrial dysfunction and could be attributed to the activation of autophagy, enhance the degradation of damaged mitochondria, and thus play a cardioprotective role (Preau et al., 2016). Another study showed that thioredoxin-1 (Trx1) had a protective role in the pathogenesis of cardiovascular disease. Electron microscopy and LC3-II/LC3-I ratios showed that autophagy appeared earlier in Trx1 overexpressing mice in the sepsis model. Trx1 overexpression prolongs antioxidant protection, attenuates mitochondrial damage, activates mitophagy and biogenesis, retains contractile reserve, and prolongs survival from sepsis (Sánchez-Villamil et al., 2016).

### Autophagy and Sepsis-Induced Acute Kidney Injury

Acute kidney injury (AKI) induced by sepsis mainly manifests as damage to the renal tubular epithelial cells, apoptosis, and elevation of creatinine. There is no uniform conclusion about the role of autophagy in AKIs of sepsis. One study indicated that activation of autophagy aggravated kidney damage in CLP sepsis model mice. After the inhibition of autophagy, the degree of AKI induced by sepsis was more serious than that of the model group (Wu et al., 2019). However, more research tends to believe that autophagy activation has a protective effect on AKIs in sepsis. Studies have found that the number of autophagosomes increased in a time-dependent manner in septic mice, but the number of autophagosomes in CLP mice decreased 24 h after CLP. Western blot and immunofluorescence also showed that the CLP induced LC3-II/LC3-I ratio increased at 6–8 h, and the number of LC3-II/LC3-I decreased significantly at 24 h. This indicates that autophagy in an AKI from sepsis is gradually slowed down. Rapamycin, which induces autophagy, can reduce renal tubular epithelial cell damage and improve renal function in the 24 h CLP model (Sunahara et al., 2018). It was also confirmed in another experiment that LC3a/b expression was induced to increase in AKIs of sepsis, which showed two peaks at 6 and 36 h after CLP. Autophagy mechanisms in the early stages of sepsis include the presence of pathogen-associated molecular patterns (PAMP) released by microorganisms or the presence of ROS, which are cytoprotective mechanisms against microbial infection. The high level of autophagy induction observed in the late stage of sepsis may be a response to a decrease in lysosomal activity, which leads to prolonged tissue inflammation and cell death. The autophagy in the late stage of sepsis may reflect an attempt of cells to adapt to lethal conditions rather than a mechanism of cell death (Karagiannidis et al., 2016). Compared with the wild-type mice sepsis model, autophagy-deficient mice exhibited more severe renal dysfunction and renal parenchymal damage after LPS injection. Moreover, analysis of kidney lysates identified higher IL-6 expression in kidney lysates from ATG7KO mice (Leventhal et al., 2016; Mei et al., 2016). Autophagy in septic kidney tissue is regulated by a variety of factors. Sirtuin3 (SIRT3) overexpression promoted autophagy in CLP mice, it up-regulates p-AMPK and down-regulates p-mTOR, attenuates sepsis-induced AKI, renal tubular cell apoptosis, and accumulation of inflammatory cytokines in the kidney (Zhao et al., 2018). Overexpression of SIRT6 down-regulates LPS-induced TNF-α and IL-6 secretion, inhibits LPS-induced apoptosis, promotes autophagy in renal tubular epithelial cells (HK-2 cells), and repairs LPS-induced renal injury. Furthermore, silencing the SIRT6 gene not only promotes TNF-α and IL-6 secretion, but also promotes apoptosis and reduces autophagy in HK-2 cells (Zhang et al., 2019b). Therefore, we still believe that autophagy plays a protective role in septic AKIs, but more specific mechanisms need to be further clarified.

### Autophagy and Sepsis-Induced Liver Injury

The liver is the main line of defense against microorganisms and the main organ responsible for sepsis-induced damage (Oami et al., 2018). Experts are currently consistent in the conclusion that autophagy is an important protective mechanism in septic liver injury. When sepsis occurs, increased autophagy can play a protective role in liver function. Studies have found that activating transcription factor 4 (ATF4) directly targets the activation of autophagy, and ATF4 expression is inhibited 48 h after LPS-induced acute liver injury. Obeticholic acid (OCA) protected mice from LPS-induced liver injury, possibly in contribution with ATF4-mediated autophagy activity in hepatocytes (Xiong et al., 2017). The lack of autophagy during sepsis may prevent the removal of hepatic mitochondrial damage and dysfunction. Another possible explanation for the lack of autophagy to aggravate liver injury and apoptosis may be its effect on endoplasmic reticulum (ER) stress and unfolded protein response (UPR) regulation (Thiessen et al., 2017). It has been reported in another study that CLP of liver-specific autophagy-deficient mice resulted in increased liver damage and inflammation. Blocking autophagy in the liver accelerated the death time of sepsis model mice. Liver autophagy protects organ failure by degrading damaged mitochondria and preventing apoptosis (Oami et al., 2018). Another study also found that increased mitophagy in sepsis had protective effects on liver function. Rg3 regulates mitophagy by activating the AMPK signaling pathway, thereby improving mitochondrial dysfunction, protecting cell and organ damage caused by sepsis. After treatment with autophagy inhibitors or AMPK inhibitors, LPS-induced mitochondrial protective function was reduced in LPS-induced human primary hepatocyte injury (Xing et al., 2017). The study found that sepsis models were replicated in young (2–3 months) and mature male mice (11–13 months) to observe liver damage, and liver damage in mature mice was higher than in young rats. Hepatocyte mitochondria functional, structural, and biological damage were associated with reduced autophagy (Inata et al., 2018). However, it should be particularly noted that increased inflammatory response was not a mechanism of hepatic injury in autophagy-deficient mice, as the extent of hepatic macrophage and neutrophil infiltration and pro-inflammatory cytokine levels were not affected by reduced autophagy. Since the number of mitochondria, mitochondrial ATP, and oxidative stress was not affected by the loss of autophagy in hepatocytes, the sensitivity of autophagy-deficient hepatocytes to LPS death is not due to the lack of hepatic mitophagy (Lalazar et al., 2016).

### Autophagy and Sepsis-Associated Encephalopathy

Studies have shown that the incidence of sepsis-associated encephalopathy (SAE) is about 35.56% (Li et al., 2016b). At 12 h after CLP, the α wave frequency of the SAE(+) group decreased significantly, the δ wave increased, the P1 amplitude decreased, and the SEP wave latency prolonged. In the SAE (+) group, the pyramidal cells were significantly reduced, even dissolved, and the cells were disordered. Significant autophagy was observed in the SAE(+) group when compared to the SAE(−) group. This suggests that autophagy may be associated with SAE (Li et al., 2016b). It is reported that cortical neurons showed an increased number of autophagosomes after 6 h of CLP, increased expression of autophagy-associated protein LC3-II, and expression of SQSTM1/P62 decline. The human IRG family member 1(IRGM1) can degrade and remove some toxins and pathogens in the body by inducing autophagy in sepsis and avoid further damage of the cells. The study also confirmed that autophagy was significantly suppressed in neuronal cells of IRGM1 knockout mice. Compared with wild-type sepsis model mice, the incidence of sepsis-induced brain injury was increased. The brain damage of IRGM1 knockout SAE(+) mice was more serious, and the number of nerve cells was significantly reduced (Huang et al., 2017). It is well-known that sustained hypercatabolism contributes to serious complications in sepsis. Sepsis also reduces the level of autophagy in the hypothalamus, accompanied by changes in high catabolism. Activation of the AMPK-induced autophagy pathway through the third ventricle injection of AICAR (AMPK activator) can effectively improve high catabolism (Cao et al., 2018). Autophagy processes in hippocampal neurons are also regulated by other cytokines, such as NF-κB. Unlike regulation in cardiac tissue (Zhou et al., 2017), the autophagy process in hippocampal neurons of septic rats might be blocked by the activation of the NF-κB signaling pathway. Inhibition of the NF-κB signaling pathway could enhance the completion of autophagy with hippocampal neurons pathological changes attenuating in septic brains (Su et al., 2015). In a mouse model of sepsis encephalopathy, immunohistochemistry showed a marked decrease in hippocampal neurons and increased glial cell infiltration after CLP surgery. Rapamycin improved cognitive impairment after sepsis by enhancing autophagy (Liu et al., 2017a). Another study on Ginsenoside Rg1 (Rg1) reached similar conclusions: impaired autophagy pathway might contribute to brain damage in SAE. This study indicated that Rg1 protected against SAE, partly through the improvement of noncanonical Beclin-1 independent autophagy (Li et al., 2017b). In conclusion, activation of autophagy is beneficial in SAE.

### Autophagy and Sepsis-Associated Myopathy

Loss of muscle mass and function caused by sepsis exacerbates the patient’s physical disuse and disability. Various factors lead to fiber atrophy through the inhibition of protein synthesis and activation of proteolytic pathways during sepsis (Stana et al., 2017). Autophagy, as an important component of the proteolytic system, is still considered controversial in the role of sepsis-induced muscle tissue damage (Morel et al., 2017). One study found that the degree of autophagy in different parts of the muscle tissue was different after CLP surgery. Compared with the diaphragm, the motor muscle is more prone to muscle mass loss caused by sepsis. This can be attributed to the sustained activation of the autophagolysosomal pathway mediated by ROS in skeletal muscle (Stana et al., 2017). However, another study suggested that activation of autophagy played a protective role in the maintenance of myofiber in sepsis. Activation of autophagy prevented the accumulation of dysfunctional organelles and toxic proteins, which can lead to muscle atrophy. In contrast, inhibition of autophagy worsened neuromuscular function (Morel et al., 2017; Chen et al., 2019). The mechanism of autophagy during the recovery phase is completely different from that in the acute phase. During the recovery of sepsis, muscle protein synthesis is increased, and with the increase in mTOR kinase activity, autophagy is inhibited. Autophagy increased 24 h after CLP, but decreased during the recovery phase, which may indicate that the need to remove damaged proteins and mitochondria had waned (Crowell et al., 2017). During sepsis, skeletal muscle protein synthesis is impaired, and proteolysis is increased to deliver amino acids to the bloodstream and supply substrates for vital organs (brains, etc.). During sepsis, supplemental amino acids antagonize the activation of skeletal muscle autophagy signals and prevent sepsis-induced muscle protein degradation (Hernandez-García et al., 2016). In summary, we believe that autophagy in sepsis is harmful to skeletal muscle.

## Autophagy and Sepsis Treatment Targets

At present, autophagy is an important self-protection mechanism for cell survival, which can maintain immune homeostasis and alleviate multi-organ failure, further improving the survival of septic animals. The protective effects of autophagy on immune cells include innate and adaptive immune responses and are involved in a variety of cellular receptors and intracellular signaling. A variety of drugs and measures have been reported to benefit septic attacks by inducing autophagy. Autophagy may be an effective target for reversing the immunosuppression of septic damage (Ho et al., 2016; Ren et al., 2017). Accelerated autophagy may reduce organ damage by modulating apoptosis (Zhang et al., 2016; Watanabe et al., 2018).

Many drugs that can improve autophagy and natural chemical monomers have been discovered, that are potential therapeutic drugs for improving the dysfunction of septic organs. Minocycline enhances mitophagy and cardiomyocyte autophagy and improves cardiac function in cardiomyocytes. The underlying mechanisms are associated with mTORC1 inhibition and mTORC2 activation (Zhang et al., 2019a). Sinomenine (SIN) is a natural alkaloid extracted from Chinese the medicinal plant Sinomeniumacutum, which may improve survival and reduce organ damage and inflammatory cytokines by partially regulating autophagy activity (Jiang et al., 2015). Artesunate AS, extracted from the Chinese medicinal plant Artemisiae Annuae Herba, inhibits LPS-induced cytokine release from macrophages by inhibiting autophagy activation (Kuang et al., 2018). Apigenin regulates redox homeostasis, and its anti-inflammatory effect may be related to its ability to control autophagy and is a potential treatment for myocardial injury in sepsis (Li et al., 2017a). Allicin can improve sepsis by inducing acute lung injury in mice by increasing autophagy levels (Peng et al., 2017). Genipin is a potential therapeutic agent for the treatment of sepsis by restoring impaired autophagy flux to prevent septic liver damage (Cho et al., 2016).

Although certain FDA-approved drugs have effects to activate or deactivate autophagy, they were not developed for the purpose to adjust autophagy. Multiple organ damage is involved in sepsis, and autophagy has different roles in different organs. Drugs that use a single activation or autophagy to protect one organ may be harmful to other organs. In addition, drugs that either activate or inhibit autophagy may have a broader spectrum of effects, such as rapamycin, which makes it more complicated to explain the role of autophagy in sepsis. Nonetheless, current evidence still suggests that some drugs could hold an exciting therapeutic potential for the control of sepsis.

## Conclusion

In this review, we discussed several physiological and pathological processes from the perspective of “autophagy” in sepsis. These views are useful to understand how autophagy maintains immune homeostasis, how it plays a protective role in sepsis-induced mitochondrial dysfunction, and how non-coding RNA regulates autophagy during sepsis. Additionally, recent studies suggested that the physiological role of autophagy could be positive and negative in some key organs. Although it has been reported that certain natural products and synthetic compounds can regulate autophagy, there is still a lack of strictly specific autophagy-modulating drugs for clinical application. Autophagy, as a conservative phenomenon in the evolutionary process, is a protective existence to the body within a certain range, but the mechanism of sepsis is complex and requires more in-depth research to reveal the role of autophagy in sepsis.

## Author Contributions

All authors participated in the writing and editing of this review article. All authors read and approved the final version of the manuscript.

### Conflict of Interest Statement

The authors declare that the research was conducted in the absence of any commercial or financial relationships that could be construed as a potential conflict of interest.
